# Apnea Detection in Polysomnographic Recordings Using Machine Learning Techniques

**DOI:** 10.3390/diagnostics11122302

**Published:** 2021-12-08

**Authors:** Marek Piorecky, Martin Bartoň, Vlastimil Koudelka, Jitka Buskova, Jana Koprivova, Martin Brunovsky, Vaclava Piorecka

**Affiliations:** 1 National Institute of Mental Health, 25067 Klecany, Czech Republic; marek.piorecky@fbmi.cvut.cz (M.P.); ma.barton@seznam.cz (M.B.); vlastimil.koudelka@nudz.cz (V.K.); jitka.buskova@nudz.cz (J.B.); jana.koprivova@nudz.cz (J.K.); martin.brunovsky@nudz.cz (M.B.); 2 Faculty of Biomedical Engineering, Czech Technical University in Prague, 27201 Kladno, Czech Republic; 3 Third Faculty of Medicine, Charles University, 10000 Prague, Czech Republic

**Keywords:** apnea, CNN, sleep EEG records

## Abstract

Sleep disorders are diagnosed in sleep laboratories by polysomnography, a multi-parameter examination that monitors biological signals during sleep. The subsequent evaluation of the obtained records is very time-consuming. The goal of this study was to create an automatic system for evaluation of the airflow and SpO${}_{2}$ channels of polysomnography records, through the use of machine learning techniques and a large database, for apnea and desaturation detection (which is unusual in other studies). To that end, a convolutional neural network (CNN) was designed using hyperparameter optimization. It was then trained and tested for apnea and desaturation. The proposed CNN was compared with the commonly used *k*-nearest neighbors (*k*-NN) method. The classifiers were designed based on nasal airflow and blood oxygen saturation signals. The final neural network accuracy for apnea detection reached 84%, and that for desaturation detection was 74%, while the *k*-NN classifier reached accuracies of 83% and 64% for apnea detection and desaturation detection, respectively.

## 1. Introduction

Sleep has been proven to play an important role in maintaining balance, preventing disease, and healing [1]. One of the most common sleep disorders is sleep apnea (sleep apnea syndrome; SAS), which is characterized by episodes of inadequate respiratory activity during sleep [2]. According to the American Academy of Sleep Medicine (AASM), sleep apnea is defined as the cessation of airflow that lasts for at least 10 s, accompanied by a decrease in blood oxygen saturation by at least 3%. Hypopnea is defined as a 30% decrease in general ventilation, with the same minimum decrease in blood oxygen saturation (3%) for at least 10 s. The most common form of sleep apnea is obstructive sleep apnea syndrome (OSAS) [1]. Research has suggested that the brain damage associated with OSAS could increase the risk of developing dementia [3,4]. Sleep apnea is described by the apnea hypopnea index (AHI), which is the average number of apnea and hypopnea episodes per hour of sleep [5]. Classification according to the AHI uses four groups: Physiological standard (AHI < 5), mild sleep apnea (AHI < 15), moderate sleep apnea (AHI 15–30), and severe sleep apnea (AHI > 30) [6]. About 2% of middle-aged women and 4% of middle-aged men are affected by the apnea disease, and the initial examination of patients is carried out using a polysomnography system [7]. This disease has a large impact on middle-aged people, who are considered the most commercially productive part of the population.

Polysomnography (PSG) is used for monitoring sleep, particularly for patients with suspected OSAS [7]. PSG can record respiratory signals (RS), electroencephalographic (EEG) data, electro-oculographic (EOG) data, electromyographic (EMG) data, electrocardiographic (ECG) signals, and pulse oximetry (PO) data [8]. In order to assist in the time-consuming scoring [9] of PSG records, efforts have been made to automate the detection of SAS [5,8,10,11]. At present, neural networks are among the most innovative and widely used classifiers in the field of biomedicine [12]. There exist methods that can accurately distinguish sleep with apnea from physiological sleep [5,13], but the automatic detection of sleep apnea events is an open topic [11]. A recent study by Zhao et al. [11] utilized a support vector machine (SVM) and a *k*-nearest neighbors (*k* -NN) model for the apnea classification problem. They also implemented a random forest (RF) model for data classification. Although studies using *k*-NN and SVM classifiers predominate, the trend of using artificial neural networks (ANN) has been carried over to the case of sleep apnea event detection [14].

There is also a laboratory [15] which is currently attempting to automatically detect apnea and hypopnea through the use of neural networks (NN) to analyze simple signals (e.g., the respiratory signal), such that they can be used in clinical practice. The trend of automating apnea detection while simplifying measurements—such that the entire PSG is not required—has been applied at various levels [16], including the use of motion sensors outside the human body [17]. Overall, apnea detection research has been shifting towards the use of machine learning, which is more flexible for working with quasi-stationary biological data [18,19]. The disadvantage of some machine learning approaches is the need to extract features. In deep learning methods (deep neural networks; DNNs), flags are extracted from a large database automatically, without the subjective choice of calculating a specific feature (e.g., amplitude or signal frequency). There are many types of DNNs; the two most commonly used types of which are the recurrent long short-term memory (LSTM) [20,21], which has an artificial recurrent neural network (RNN) [22] architecture, and convolutional neural networks (CNNs) [23]. These can be applied to various signals, such as ECG [24,25], airflow in the nasal cavity [26,27], EEG [14], or chest movements [28].

As not all published methodologies have publicly available software, and as no appropriate implementation had an interface that would be usable for physicians in our sleep laboratory, we designed, tested, validated, and implemented a convolution neural network with a graphical interface. The main goal of our research was to create automatic software for detecting periods of possible sleep apnea or hypoponea for future use in our sleep laboratory (it is also free for use in other laboratories). The proposed methodology imitates decisions made by a physician, according to American Academy of Sleep Medicine (ASSM) scoring [6]. This software can alert physicians to areas of interest and speed up the process of scoring PSG recordings.

## 2. Materials and Methods

A large database of PSG data was created for the most effective training, testing, and validation. The implementation itself was written in a Python programming environment. This chapter describes the PSG data set, its pre-processing and design, and the training and validation of the neural network.

### 2.1. Data Acquisition

Data were obtained in the sleep laboratory during a standard PSG examination. A BrainScope device (M&I spol. s.r.o., Prague, Czech Republic) with a band-pass filter of 0.1–200.0 Hz was used. The records were obtained with a sampling frequency of 1 kHz, and the records were down-sampled to 250 Hz by decimation, with decimation factor equal to 4. The recordings included 19 EEG channels, 2 EOG channels (horizontal and vertical), 3 EMG channels (1 from the chin, 2 from the lower limbs), airflow through the nasal cavity, 2 movement channels located on the chest, and an oxygen saturation channel (SpO${}_{2}$). As only the airflow and SpO${}_{2}$ signals were used to detect apnea and desaturation, each signal was down-sampled to 50 Hz by simple decimation. The approximate length of each record was 8 h. Data were scored by sleep physicians, according to the AASM standard [6]. Scoring included marking individual phases of sleep as well as, for example, sleep events or limb movements. Tags summarizing apnea-related events are listed in Table 1.

The original data set contained 800 anonymized PSG records. For technical reasons, 333 records were excluded. The most common reason was a missing scoring or the presence of odd marks (the event in the record had only a beginning or an end), and it was not possible to correctly identify the marked sections without the intervention of a somnologist. As a result, the used database contained 255 records suitable for processing simultaneous oxygen saturation channels and nasal airflow, and 477 records suitable for processing nasal airflow only.

The number of apnea events varied across records. This imbalance could result in bias in favor of fewer individuals with a high incidence of apnea events, and the network would not be sufficiently generalized. For this reason, Tukey’s method was used to limit the maximum number of segments from one individual. The confidence coefficient was set to 1.5 [29]. The individual records also contained a large difference between the number of segments with apnea and those with normal breathing. For example, a patient with AHI = 75 would have 2.5 h of apnea over a total of 8 h record. Even with such a high AHI score, only 31% of the segments would be apnea segments. When training a CNN, it is appropriate to work with data in a ratio of 1:1 (in this case, apnea:physiological breath) [30]. For the apnea segments, segments of normal breathing were randomly selected to maintain this ratio.

### 2.2. Data Pre-Processing

**Figure 1:**
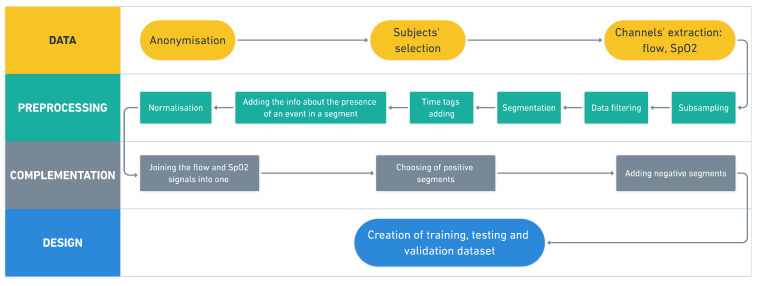
Block diagram of pre-processing pipeline.

The data pre-processing was divided into four stages, see Figure 1. First, the data were prepared (see the yellow part of the diagram). Second, the data were pre-processed, including sub-sampling, filtering, and/or normalization (see the green part of the diagram). Third, the data were complemented (see the gray part of the diagram) and, finally, the data were designated into training, testing, and validation data sets (see the blue part of the diagram). Two signals (airflow and SpO${}_{2}$) were chosen for consequent analysis. These two signals were preprocessed separately (the green part of the diagram), then were connected into one block for classification. Figure 1 shows the whole process of the data processing.

**Figure 2:**
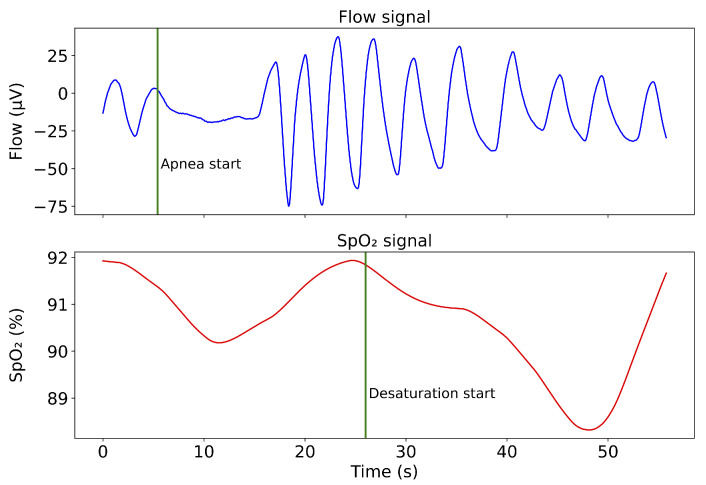
Demonstration of SpO${}_{2}$ decline response in apnea.

Numerical filtration of the airflow suppressed frequencies above 5 Hz and smoothed the signal; for this purpose, a Butterworth IIR filter (order 10) was applied. The phase shift of the designed filter has a linear characteristic in the range from 0 to 50 Hz. SpO${}_{2}$ was originally sampled with a period of two seconds. A quantization step of 1% ensured a stepped signal shape. After down-sampling to 50 Hz, oscillations were observed at sharp transitions. To suppress and smooth the signal, an IIR Butterworth filter (order 2) of the low-pass type with a cut-off frequency of 0.02 Hz was applied. Signal segmentation is performed using a sliding window of 10 s with an overlap of 90% (9 s). The window length was chosen, based on the AASM definition, as the minimum length to indicate the apnea segment. The size of the overlap was chosen according to a previous study [31]. To unify the signal amplitude across patients, a Z-score was applied to the partial records. It was necessary to take into account the delay in the SpO${}_{2}$ signal; see Figure 2. This is a physiological delay [32], which averages 25 s. For this reason, the SpO${}_{2}$ signal was shifted by 25 s.

The last pre-processing step was division of the data into training, test, and validation sets. An NN is taught using a training set, tuning is performed using a validation set, and the testing data set is utilized to evaluate the functionality of the network. The data were divided in the ratio 2/3:1/6:1/6 (training:validation:testing). The division took place at the record level, such that the NN was trained on a different data set than the one on which the subsequent testing is performed. The final size of the data set for apnea detection is seen in Table 2, and that for desaturation detection in Table 3. The number of segments for desaturation is larger, as the decrease in SpO${}_{2}$ not only causes apnea, but also hypopnea (not included in apnea).

### 2.3. NN Design

The use of deep neural networks for data classification has become popular. However, there are many variants of NNs, among which CNNs are often used. These networks make it possible to easily recognize individual elements in signals.

An NN consists of various different layers. In a CNN, the convolution layer is one of the main layers, in which the mathematical operation of convolution is applied to the input signal. Moreover, 2D convolution was used in this case, with one of the parameters being the number of individual filters. This number theoretically corresponds to the number of symptoms being searched for. A max-pooling layer usually follows each convolution layer, which reduces the size of a given data space. This reduction ensures that the next convolution layer has different data input and can search for more general features. The regularization layer is located before the next convolutional or fully connected layer (dense layer). Its task is to generalize, thus preventing over-learning the network on a given training set. The basic method for regularization is called dropout, which randomly turns selected neurons and their connections on and off during testing [33]. In the Tensorflow version 2.0 library, a dropout value of 0 means that a neuron will never be discarded. The dense layer usually follows, as the last layer. Its task is to convert all feature maps created by the convolution layers into a 1D vector, and it learns to choose the right combination that best suits the segment. The number of neurons in final layer was set to 1.

**Figure 3:**
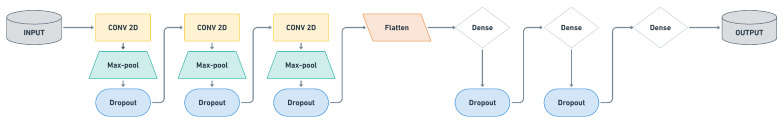
Visualization of the optimal NN design for apnea detection.

A CNN consisting of six layers was chosen, with regard to the results of previous studies [26,27]. The visualization of the NN is depicted in Figure 3.

#### 2.3.1. Setting of Parameters

Two types of activation function were used in this study: namely, the ReLU function [34] and the sigmoid function. The sigmoid function was used in the output layer and the ReLU function was used in all of the hidden layers. The loss function used binary cross-entropy with sigmoid activation [35], the batch size was set to 1000, and the Adam algorithm was used as an optimizer [36].

#### 2.3.2. Hyperparameter Optimization

**Figure 4:**
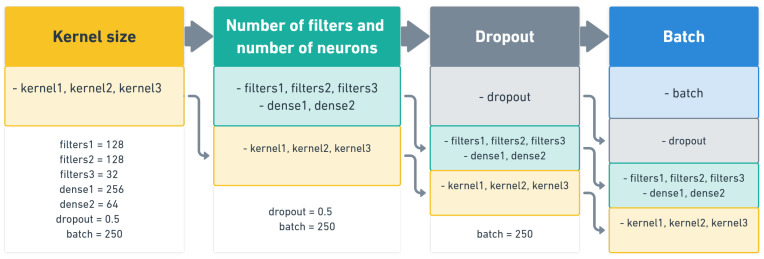
Principle scheme of hyperparameter optimization.

The grid search method was used for hyperparameter optimization. This method is based on defining specific hyperparameter values and testing the best results under a concrete hyperparameter combination [37]. The principle scheme of hyperparameter optimization is depicted in Figure 4.

The first-tested hyperparameter was the kernel size, assuming that ideal kernel is not the same in each CNN layer. The specific values of kernel size for each of the layers are defined in Table 4. The other hyperparameters were set as fixed, for testing purposes. These parameters are defined in Table 5. The batch size was set to 250 and the number of epochs was set to 50.

The kernel size parameter is independent of the segment number, so the kernel size parameter was tested on a data set consisting of 100,000 segments of the original data set. Thus, it was basically trained on 68,298 segments, after dividing into training, testing, and validation sets.

The second-tested hyperparameters were the number of filters for convolutional layers and number of neurons in fully connected layers, which were tested simultaneously. The tested values are described in Table 6. The other hyperparameters were set as fixed for testing purposes. These parameters are defined in Table 7. The batch size was set to 250 and the number of epochs was set to 50.

It was observed that the best-performing structures significantly differed in kernel and layer sizes. As mentioned above, the grid search was conducted on subsets of the original training set. It was assumed that the optimal kernel sizes and sizes of layers were subject- and testing set-specific. Thus, three best combinations of hyperparameters were averaged, in order to facilitate the higher generality of the network. A number of averaged networks was chosen intuitively, based on observed variations of the hyperparameters in the sorted list of networks.

The tested values for the dropout hyperparameter were in the range of 0.0 to 0.8 in 0.1 steps. The batch size was set to 250 for testing purposes. This hyperparameter was tested on the whole training data set.

The batch size hyperparameter was examined last. Its tested values were as follows: 100, 250, 500, 1000, and 2000.

### 2.4. Evaluation

The parameters that we focused on in the design phase of the network were accuracy and error. ROC analysis (considering specificity and sensitivity) was used to select the appropriate threshold in the final classification. As most studies compare their proposed networks to other standard automatic classifiers (often *k*-NN), we compared our network through assessing the differences in the classification results between the proposed neural network and a *k*-NN classifier. Neural networks are adapted to a specific signal, which makes it harder for direct comparison. In our case, we used a less-common variant of the combination of breath and SpO${}_{2}$, as it imitates a physician’s decision based on the ASSM standard. The *k*-NN method is a very simple and easily reproducible algorithm, which has been widely used in past studies as a reference solution to the apnea detection problem. The nearest neighbors were estimated based on the Euclidean distance.

### 2.5. GUI for Physician

A graphical user interface (GUI) was created using the AppJar library (available at www.appjar.infoi, accessed on 1 September 2021). Python 3.6+ and the following libraries are required for proper functionality: Keras 2.3.1, Tensor ow 2.0.0, AppJar 0.94.0, Numpy 1.18.0, Scipy 1.4.1, Struct, Window slider 0.8, Collections, Pytictoc, Shutil. All of these libraries are available from https://pypi.org/, accessed on 1 September 2021.

## 3. Results

The results of this work are divided into two parts: The first part provides the results of the design of the CNN and its parameters, while the second part contains the results of apnea detection using the implemented network, in comparison with the *k*-NN classifier.

### 3.1. Designed CNN

#### 3.1.1. Hyperparameter Optimization

**Figure 5:**
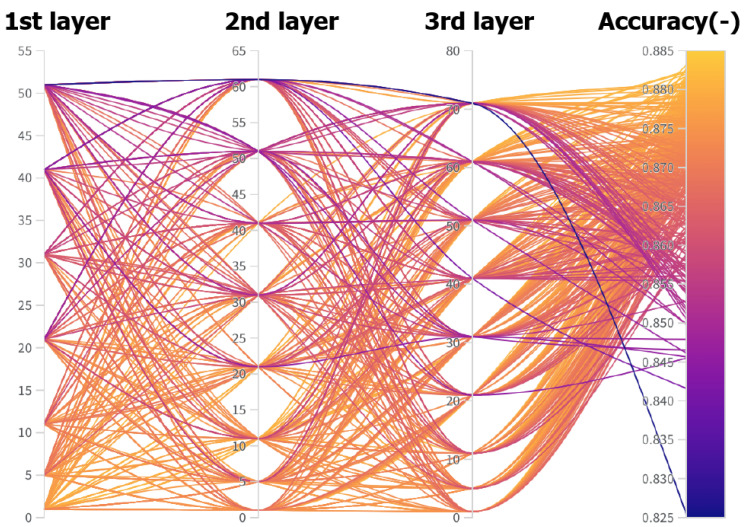
Parallel graph of all combinations and their results, in terms of accuracy. Generated using the Weights and Biases software.

To determine the ideal kernel size, 416 combinations with a length of 50 epochs were randomly tested. Figure 5 presents the individual combinations.

In Table 8, the five best combinations, selected according to training accuracy, are given. These results show a very high similarity. As such, their arithmetic mean was used to define the resulting dimensions of the layers. The resulting layer dimensions were: 1, 15, 45. If the convolution kernel length is 1, the corresponding layer provides simple scalar multiplication.

**Figure 6:**
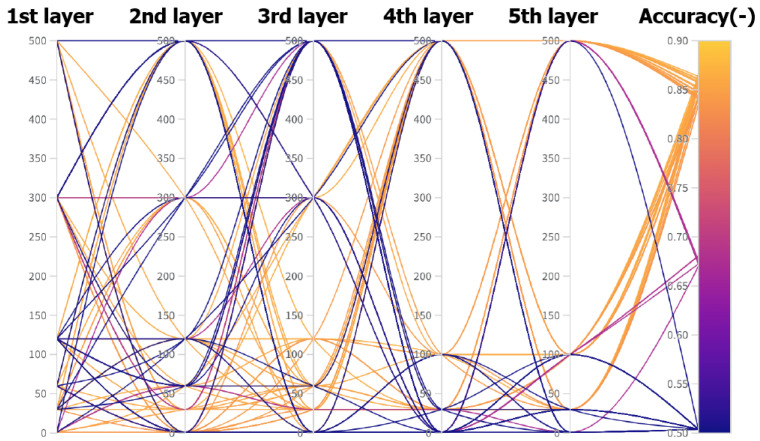
Parallel graph of all combinations of number of neurons in dense layers and number of filters in convolutional layers. Generated by the Weights and Biases software.

To identify the ideal number of neurons in the dense layer and the number of filters for the convolutional layers, we calculated 79 random combinations. Figure 6 shows the evaluation of the tested combinations.

Table 9 shows the first five best combinations (sorted by accuracy on the training set). The final values for the numbers of filters and neurons were chosen as an arithmetic mean of these five best combinations. The number of neurons in the dense layers were 148 and 86 (fourth and fifth dense layers, respectively). The number of filters for each convolution layer were 174, 308, and 96 (first, second, and third convolution layers, respectively).

**Figure 7:**
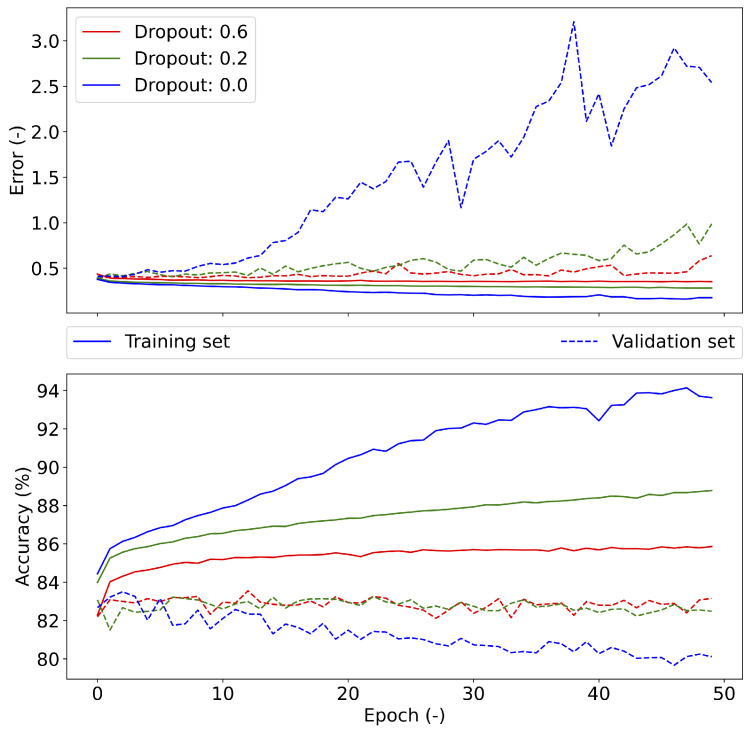
Different dropout settings and their results in the training and validation of the CNN.

**Figure 8:**
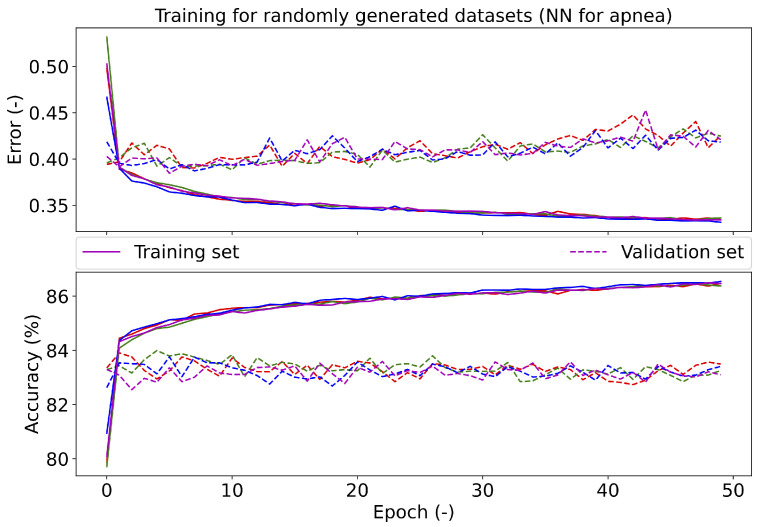
The course of testing the final neural network for apnea detection. Different colors represent different divisions into training and validation data sets.

The dropout was tested in the range of 0.0–0.8 in 0.1 steps. The highest validation accuracy of 83.16% was achieved with a value of 0.6; see Figure 7, which shows the error and accuracy of the set validation when using different dropout values. The resulting dropout was selected to be 0.6, as the error in the validation set approached the error values on the training set (red solid and dashed lines in Figure 7). A larger dropout value was not used, as the accuracy on the training set decreased above 0.6. At the same time, the validation set had the same trend for a dropout value of 0.2 and the selected value of 0.6. For apnea investigation (see Figure 8), the error tends to increase with a higher number of epochs on the validation set. The accuracy does not change on the validation set after reaching the break. For desaturation, the trend error and accuracy were the same for the training and validation sets. The best batch size was 1000, due to the accuracy (83.29%) on the validation data set; see Table 10.

The optimal kernel size was defined based on the grid search method. Additional testing for different kernel sizes in the first layer was performed. Table 11 represents the results of additional testing.

#### 3.1.2. Final Design of the Proposed CNN

Based on the hyperparameter optimization, the final structure of the CNN was implemented. The design and parameters of the network are shown in Table 12, where *K* is the size of the convolution kernel, *F* is the number of filters, *M* is the size of maxpool kernel, *D* is the dropout value, and *N* is the number of neurons.

### 3.2. Apnea Classification Results

The results of the sleep event detector were calculated on a testing data set, which always contained complete records of subjects. The same testing set was subsequently used for detection using the standard *k*-NN method. The results were evaluated using ROC analysis.

#### 3.2.1. Results of CNN Based Classification

**Figure 9:**
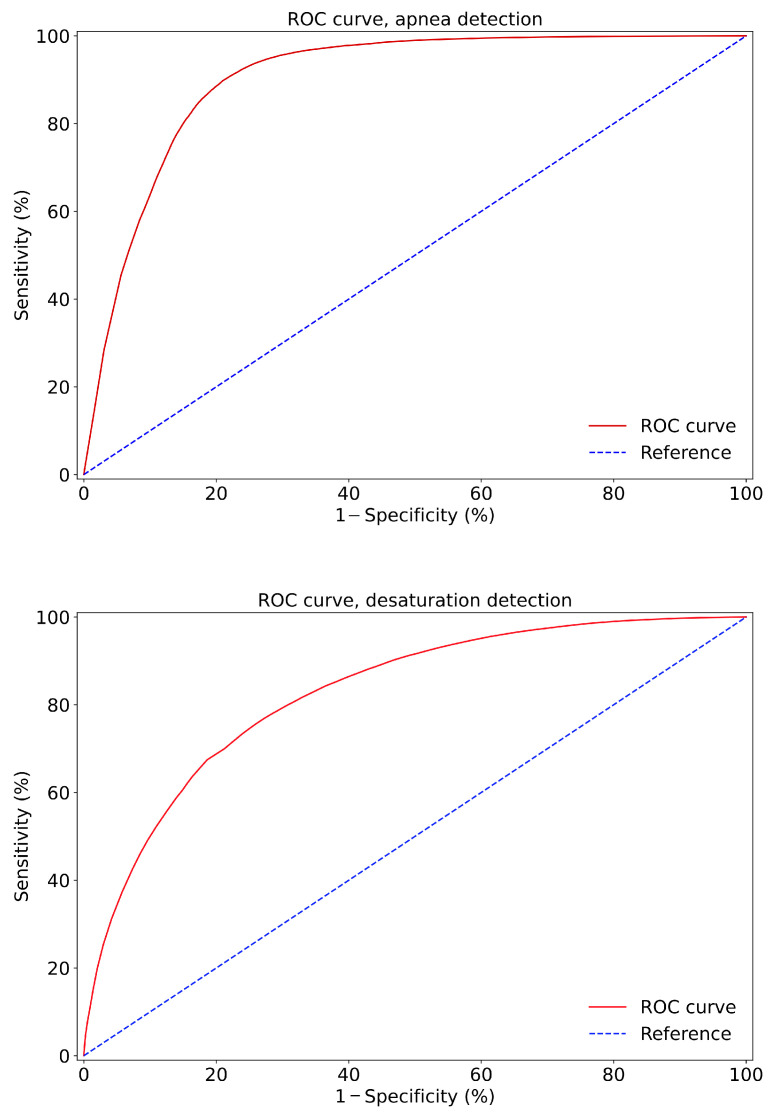
ROC curve for apnea (**top**) and desaturation (**below**) segment analyses.

The results of apnea segment classification using the deep neural convolutional network are shown in Table 13, under various selected thresholds. The results of desaturation segment classification are included in Table 14. The graph in Figure 9 shows the ROC curve, which describes the relationship between the sensitivity and specificity, depending on the threshold values, in the case of apnea and desaturation detection.

#### 3.2.2. Results of *k*-NN Based Classification

According to Tukey’s method, the maximum limit for the number of segments with an apnea event was determined for the creation of the data set (i.e., 559 segments). To reduce the time demands of the *k*-NN classification method, principal component analysis (PCA) and the t-distributed stochastic neighbor embedding (t-SNE) were used to reduce the data dimensionality before *k*-NN classification. The accuracy for different settings of *k* in the *k*-NN method on the reduced validation set (50,000 segments) is shown in Table 15 (left). The most suitable coefficient for apnea detection was k=20, while that for desaturation detection was k=500. The sensitivity and specificity results for this setting are presented in Table 16.

## 4. Discussion

Deep learning techniques have been increasingly used to diagnose sleep apnea [38], including convolutional neural networks, such as was used in our study. As in a large part of neurodiagnostic methods, the problem is the small number of measured subjects (i.e., small data set size). The main benefit of this work can be considered to be the training of a deep convolutional network on a large database. PSG records were obtained with a sampling frequency of 1000 Hz. Such a high frequency is due to the acquisition of EEG recordings, in which it is necessary to evaluate brain frequencies up to 30 Hz as standard. Only airflow and SpO${}_{2}$ signals were processed to detect apnea and desaturation in this study. Therefore, it was possible to down-sample these signals. The shapes of the airflow and SpO${}_{2}$ signals are similar to a sinusoid with a frequency of about 0.2 Hz. It was therefore possible to sample at a frequency of 50 Hz. The signal was down-sampled five times, which significantly reduced the computational complexity and memory usage without loss of information. Even so, the design and training of the network were very time-consuming; for example, to determine the ideal number of neurons in fully connected layers and the number of filters for convolutional layers, 79 random combinations were calculated. In total, the calculation took 92 h. Additionally, hardware limitations prevented testing a batch size larger than 2000, as the used computing unit had limited parallel computation ability.

We designed a neural network and then performed its learning, validation, and testing stages. Thanks to the use of a large database, we adapted the network and its parameters to detect apnea. Pre-trained networks have also been used in the literature [39], but we still assume that the greatest effectiveness can be obtained by training for a specific pattern (in this case, apnea). The baseline approximate neural network depth was determined from a previous study [31]. The parameters of the NN were further estimated to achieve the highest validation accuracy.

The hyperparameters of CNN were estimated separately, not taking into account the fact that some parameters depend on each other; however, if the parameters were tested as dependent variables, it would not be possible to evaluate their effect separately. For the desaturation detection, the same NN design with the same hyperparameters as in the case of apnea detection was used, based on the knowledge that the analyzed signals were generated from same biosignals. For future research, it may be interesting to test and optimize the hyperparameters for each of the events separately.

The resulting dimensions of the convolution kernel were 1, 15, 45. Gradual enlargement of the convolution kernel is an expected phenomenon, which guarantees that features of different lengths gradually flow between layers. Our test results indicated that a size of 1 for the first layer kernel gave the best results; see Table 11. It is possible that this layer could be replaced by a simpler layer in the future, which may speed up the calculation and the detection itself. The batch size parameter was set to 2000. A larger batch size decreases the accuracy of a NN on the training data set [40]. In our case, the use of a larger batch ensured greater regularization and, thus, greater accuracy on the validation set. A higher batch size could not be tested, due to hardware limitations.

**Figure 10:**
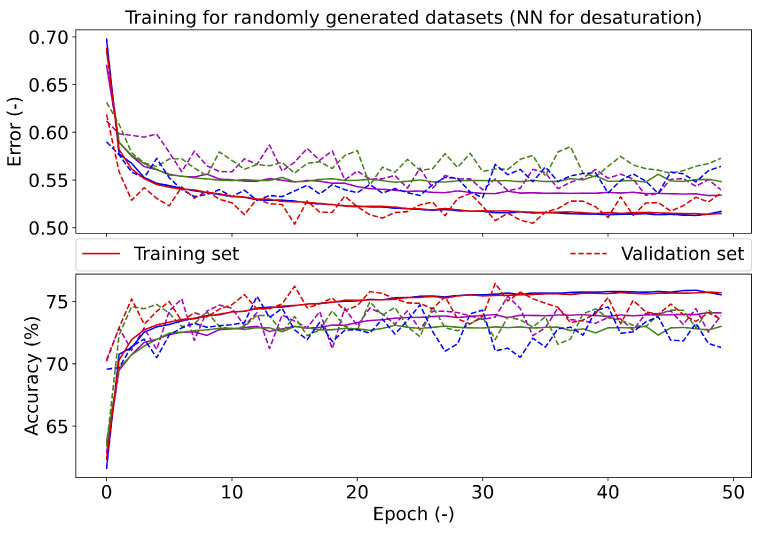
The course of testing the final neural network for desaturation. Different colors represent different divisions into training and validation data sets.

The training of the NN was performed four times on randomly generated training, validation, and testing data sets. The graph in Figure 8 shows the course of the training of the proposed NN for apnea detection. According to the results, it can be concluded that the NN had similar character for the different data sets (different curve colors). The graph in Figure 10 shows the course of the training of the NN for desaturation detection. The accuracy and error had similar characters when comparing the training and testing phases, so it should be easy to train the NN for desaturation detection.

During the training of the proposed CNN for apnea detection, an increase in the validation error was observable, which increased with the steps of the training while, at the same time, the validation accuracy decreased. This indicates the over-fitting of the neural network. In order not to implement an over-fitted network, the method of early interruption of learning was used. The final epoch was not included but, instead, the one that had the smallest validation error during training was used. For the network trained for desaturation classification, the setting with the smallest validation error was selected. The final accuracy of the designed neural network was 84% for apnea detection and 74% for desaturation detection. For the *k*-NN reference method, the accuracy was 83% in the case of apnea detection, while that for desaturation detection was 64%; that is, both were lower than those of the proposed CNN. Studies comparing the results of classification using *k*-NN and ANNs vary in the difference between the accuracy of these classifiers (accuracy of ANN minus *k*-NN). For example, some studies [14,41,42] have described a positive difference (i.e., higher accuracy for the ANN) of 9.7% on average. In contrast, other studies [43,44] have reported a negative difference (i.e., higher accuracy for *k*-NN) of 2% on average. Furthermore, the study by Mendel et al. [45] reported zero difference (identical accuracy for both methods). When detecting desaturation, the difference in our accuracy study was similar to that of [14,42]. Although the *k*-NN classifier is much simpler, compared to the ANN, the accuracy on the testing data set of *k*-NN (83%) was similar to that obtained by the ANN (84%) in our case; this was also comparable with the previous papers. This result ensured us that the *k*-NN is a meaningful reference method for ANN-based classifiers. Obviously, detection using a deep neural network has a great advantage over the use of a *k*-NN classifier, in terms of the significantly lower time requirements of the detector. Apnea detection using CNN processed up to 8 h of recording in 36 s, while using *k*-NN required 45 min (as measured on an AMD Ryzen 9 3900X processor). Calculating *k*-NN with high-volume data is very demanding. With a lower class processor (i.e., standard sleep department equipment), the calculation would take even longer. This is an essential parameter for practical implementation in a clinical setting. The disadvantage of using a neural network, in terms of hardware, is that it requires a processor that supports the Advanced Vector Extensions (AVX) instruction set. AVX support has been introduced for most processors since 2013; so, for implementation in practice, it is necessary to install the proposed detector only on devices containing such processors, which do not have to be in all sleep labs.

For the purposes of training, as well as the detection itself, the original continuous signal was segmented. For subsequent display by the software used in the sleep laboratory, it is necessary to convert the segments back to a continuous signal after classification. Each segment containing 80% of the event in a sequence of several such segments in a row (positive) indicates an event (apnea/hypopnea). In the study by Choi et al. [31], valid apnea events were considered as five consequent events in row. We also differ from other studies, in that we detected apnea with an accuracy of 1 s (10 s window with a bias of 90%). For example, in the study by Varady et al. [19], a 16 s window was used without an overlay, such that they detected apnea with an accuracy of 16 s. Furthermore, in the study by McClure et al. [46], a 15 s window was used, also without an overlay.

The event detection process takes place on data with the same pre-processing as was used to learn the CNN. Apnea and desaturation detection are performed separately, and the detected events are saved to a newly copied file. The output of a neural network is the probability that a given segment contains an event. This allows the threshold to be selected, according to whether greater specificity or sensitivity is desired.

Various biological signals can be used to identify apnea. Neural networks have been applied to EEG [14], EKG [47], respiratory [48], and SpO${}_{2}$ [49] signals. Apnea detection is often performed using random forest, support vector machine, and *k*-NN methods, which have accuracies of about 80–90% [7]. Some new studies have reported high classification accuracy (sensitivity and specificity >90%), but the data sample was very small (17 subjects) [50]. Our proposed NN had lower classification accuracy, but we trained and tested the NN on a data set, which was 15 times larger. Some previous studies have used an existing NN topology and used transfer learning to recompute the ideal parameters; for example, Singh et al. [51] have used the AlexNet NN with different types of classifiers. Table 17 summarizes the results of apnea detection in the case of different classifiers and used signals in the previous studies.

Note that the SpO${}_{2}$ and nasal airflow signals were used in our study to detect apnea, in order to refer to the standard scoring procedure of the AASM scoring manual. Studies which specifically used the SpO${}_{2}$ or nasal airflow as reference signal resulted in accuracies ranging from 79.6% to 97.64%; see Table 17.

The highest accuracy was obtained in the study by Mostafa et al. [49]; namely, 97.64%. The deep belief network (DBN) classifier was used in this case, and the SpO${}_{2}$ reference signal was used. The first two layers of the DBN classifier were constructed using restricted Boltzmann machines (RBM), and the final layer was a softmax layer. The methodology was tested on two publicly available data sets consisting of 32 and 25 records, respectively. The structure of the DBN was fixed, and the hidden layer neurons were only optimized for the UCD database. The same DBN classifier was used on different data set, and the accuracy decreased to 85.26%. Lower accuracy values can be reached when different data sets are used for training and testing. In the first case, of training and testing on the same data set, the accuracy values were higher than in proposed methodology. On the other hand, the sensitivity reached lower values in the study of Mostafa et al. [49]. In case of testing on a different database, the accuracy was comparable, and the sensitivity was lower than those reported in our study. Furthermore, AUC values were not presented in the study by Mostafa et al. [49].

In the study by Biswal et al. [21], SaO${}_{2}$, airflow, and signals from the chest and abdomen belt were used as reference. Two publicly available data sets were used in this case. They utilized an RCNN for classification. Different accuracies were reached in the case of training and testing on the same data set; namely, 85.7% and 81.7%, respectively. When testing on different data sets, it decreased to 78.7% in the first case while, in the second case, the accuracy increased to 83.3%. This study used more reference signals than in the proposed methodology, with comparable results.

In the study of Pathinarupothi et al. [20], SpO${}_{2}$ and instantaneous heart rate (IHR) were used for apnea classification. The LSTM method was used in this case. The accuracy when using SpO${}_{2}$ reached 95.5% and, in the case of using a combination of SpO${}_{2}$ and IHR, the accuracy decreased to 92.1%. This study proposed minute-to-minute apnea classification. It involved the analysis of 35 subjects but, in the case of the SpO${}_{2}$ reference signal, it involved only 8 subjects. In comparison to the proposed methodology, the study of Pathinaupothi et al. [20] was performed on a data set which was approximately 30 times smaller, and with 60 times smaller time resolution.

In a study by Cen et al. [28], the 2D CNN method was used for apnea detection, based on the SpO${}_{2}$, oronasal airflow, and movements of ribcage and abdomen reference signals for classification. The study was performed using 25 patients. The method was based on feature extraction, and the concrete features were validated by the CNN. An average accuracy value of 79.61% was reached across all classes; however, the average classification accuracies in normal, hypopnea, and apnea classes were 82.20%, 53.61%, and 66.24%, respectively. In comparison to the proposed methodology, the study by Cen et al. [28] was performed on a smaller data set, with lower accuracies and smaller time resolution; however, the study by Cen et al. [28] was designed on a data set based on more reference signals than our proposed methodology.

The study by Mostafa et al. [52] used an SpO${}_{2}$ reference signal for classification by a 1D CNN. Three publicly available databases were used, consisting of the records of 8, 25, and 70 subjects, respectively. The largest database was used for training purposes, and then the model was tested on the others. This study also performed a transfer learning technique to retrain the CNN on the other databases. All of the tests were performed considering 1 min-, 3 min-, and 5 min-long segments. The accuracy was in the range of 84.53–92.65% in the first case, and the sensitivity was in the range 56.72–91.64%. Lower values were reached in the case of testing on different databases than the training set. In the second case (i.e., transfer learning), the accuracy values were in the range of 84.85–94.24%, and the sensitivity was in the range 93.32–96.78%. Therefore, transfer learning improved both the accuracy and sensitivity. In comparison to the proposed methodology, the study of Mostafa et al. [52] was performed on a smaller data set, with comparable accuracies and smaller time resolution. The sensitivity was lower in the study by Mostafa et al. [52]. The study by Mostafa et al. [52] used only one reference signal; namely, SpO${}_{2}$.

Overall, the use of ECG signals generally corresponds with high apnea classification accuracy. On the other hand, in the study of Pathinarupothi et al. [20], the success of the classification decreased with the use of the ECG signal. In our manuscript, the proposed solution was to mimic the work of a physician who scores apnea according to ASSM standards.

A large number of approaches have been tested to detect apnea; however, a major limitation of most studies is the size of the used data set. For example, Varady et al. [19] conducted research on a set of 18 PSG recordings, but with an average recording time of only 4 h. The study of Almazaydeh et al. [53] had a sample of 50 subjects, but these were healthy individuals who consciously simulated respiratory pauses. In the study of Janbakhshi et al. [54], 35 records were used. Probably the most extensive study we were able to find is that of Steenkiste et al. [48], who used a data set [55] (available online at https://physionet.org/content/shhpsgdb/1.0.0/, accessed on 2019) from which they selected 2100 individuals, of which 100 individuals were used for training, and 2000 for testing. However, this is a data set [55] from 1997, where PSG is presented as a “home” variant and apnea was marked from saturation and EEG; the primary orientation of the data set is on cardiovascular disease.

A limitation of the study was the lower accuracy than the highest transmission mentioned in the current literature. The lower portability was due to the effort to imitate the ASSM manual while, at the same time, streamlining the network in the context of minimizing network dispositions. The established network was tuned within a limited number of iterations, so it is possible that a higher accuracy could be achieved. At the same time, unlike other studies, we did not use segment-level information (from each patient) to train the network, instead focusing at the record level from each patient. Subsequently, our network obtained information from new patients (the network has no previous information), and we presented the accuracy of the classification on this approach. Another limitation lies in the usage of only the *k*-NN reference method to assess the performance of the proposed CNN. Further work can be carried out to interpret the weights across kernel layers and to bring insights into the features differentiating respiration events from the baseline. The current paper relied on the scoring guidelines, and more information about the respiration events could be obtained from the trained network. In the case of finding the optimal kernel length, the number of filters, and the number of neurons, it was observed that the best-performing structures only slightly differed in their accuracy. For this reason, a mean value was used. It is possible that some other metric may be more suitable for identifying the best solution. We presented how the convolutional network behaved when training on such a large database. In addition, we offer open access to our code for further use. The proposed approach mimics the work of a physician, such that it is possible to use the methodology for the preparation of training for physicians and laboratory nurses in sleep laboratories. Another advantage is the high time resolution that the proposed network offers.

**Table 17:** Comparison of different types of classifiers and their accuracies.

Ref.	Signal	Classifier	Accuracy	Results Sensitivity	Specificity
[21]	CB, AB, SaO${}_{2}$, AF	ut. RCNN	85.7%/81.7%	-	-
[21]	CB, AB, SaO${}_{2}$, AF	ut. RCNN	78.7%/83.3%	-	-
[49]	SpO${}_{2}$	DBN	97.64%	78.75%	95.89%
[49]	SpO${}_{2}$	DBN	85.26%	60.36%	91.71%
[20]	SpO${}_{2}$, IHR	LSTM	92.1%	84.7%	-
[20]	SpO${}_{2}$	LSTM	95.5%	92.9%	-
[28]	SpO${}_{2}$, ONAF, RIB, AB	CNN2D	79.6%	-	-
[52]	SpO${}_{2}$	CNN1D	84.53–92.65%	56.72–91.64%	90.19–94.60%
[52]	SpO${}_{2}$	CNN1D	84.85–94.24%	58.32–92.04%	93.32–96.78%
[31]	NP	CNN	96.6%	81.1%	98.5%
[27]	THO	CNN	70.7%	-	-
[27]	ABD	CNN	72.3%	-	-
[27]	NAF	CNN	77.6%	-	-
[27]	THO, ABD	CNN	77.7%	-	-
[27]	NAF, THO	CNN	82.0%	-	-
[27]	NAF, ABD	CNN	82.6%	-	-
[27]	NAD, ABD, THO	CNN	83.5%	-	-
[26]	NAF	CNN	74.70 ± 1.43%	-	-
[24]	ECG	CNN	98.91%	97.82%	99.20%
[56]	ECG	AB	87.33%	81.99%	90.72%
[57]	ECG	LS-SVM	84.74%	84.71%	84.69%
[44]	ECG	BA	85.97%	84.14%	86.83%
[58]	ECG	SLBP	89.80%	88.46%	90.63%
[59]	ECG	1-D CNN	87.90%	-	-
[60]	ECG	CNNLSTM	86.25%	-	-
[51]	ECG	AlexNet CNN	86.22%	90.00%	83.82%
[14]	EEG	*k*-NN	75%	70%	92%
[14]	EEG	SVM (RBF)	95%	90%	100%
[14]	EEG	SVM (polynomial)	99%	100%	98%
[14]	EEG	ANN	86%	75%	100%
[5]	EEG	DFA + SVM	95.1%	93.2%	98.6%
[10]	EEG	CNN + LSTM	76.5–84.5%	-	-

## 5. Conclusions

A sleep apnea and desaturation detector were constructed, based on a deep convolutional neural network. The Python language and TensorFlow library were used for the implementation. The hyperparameters of the CNN were optimized for the case of apnea detection, and the CNN was then trained for apnea and desaturation detection. After optimization and evaluation of the CNN parameters, a comparison with the *k*-NN method, in terms of detection capability, was performed.

The accuracy of the resulting neural network on the test data set, for apnea detection, was 84%, while that for desaturation detection was 74%. In comparison, using *k*-NN, the accuracies were 83% and 64% for apnea and desaturation detection, respectively. From a practical point of view, calculation using a CNN is faster and provides the possibility of using an optional threshold that changes the resulting sensitivity and specificity, according to the user’s preferences.

## Figures and Tables

**Table 1:** Example of marker identification and their number from real PSG.

Tag ID	Dfile Tag	Tag Numbers	Dfile Text
130	O+	225	start OSAS
131	O−	225	stop OSAS
132	A+	10	start CAS
133	A−	10	stop CAS
138	D+	159	start desaturation
139	D−	159	stop desaturation

**Table 2:** Numbers of segments and subjects in data sets for NN apnea detection.

	Number of Segments	Number of Subjects
Training data set	351,550	175
Validation data set	87,180	38
Testing data set	88,882	42

**Table 3:** Numbers of segments and subjects in data sets for NN desaturation detection.

	Number of Segments	Number of Subjects
Training data set	777,640	174
Validation data set	193,886	36
Testing data set	198,082	45

**Table 4:** Tested values for convolutional layer kernel sizes.

Layer	Variable	Values
First	kernel1	1, 5, 11, 21, 31, 41, 51
Second	kernel2	1, 5, 11, 21, 31, 41, 51, 61
Third	kernel3	1, 5, 11, 21, 31, 41, 51, 61, 71

**Table 5:** NN structure for kernel size optimization.

Layer Name	Parameter
2D CL	K (1 × kernel1 ), F 128
MaxPool	M (1,2)
Dropout	D 0.5
2D CL	K (2 × kernel2), F 128
MaxPool	M (1,2)
Dropout	D 0.5
2D CL	K (1 × kernel3), F 32
MaxPool	M (1,2)
Dropout	D 0.5
Flatten	
Dense	N 256
Dropout	D 0.5
Dense	N 64
Dropout	D 0.5
Dense	N 1

**Table 6:** Tested values of filter number and number of neurons.

Layer	Variable	Tested Values
First, convolutional	filter1	1, 30, 60, 120, 300, 500
Second, convolutional	filter2	1, 30, 60, 120, 300, 500
Third, convolutional	filter3	1, 30, 60, 120, 300, 500
Fourth, fully connected	dense1	1, 30, 100, 500
Fifth, fully connected	dense2	1, 30, 100, 500

**Table 7:** NN structure for optimization of number of filters and number of neurons.

Layer Name	Parameter
2D CL	K (1 × 1), F filters1
MaxPool	M (1,2)
Dropout	D 0.5
2D CL	K (2 × 15), F filters2
MaxPool	M (1,2)
Dropout	D 0.5
2D CL	K (1 × 45), F filters3
MaxPool	M (1,2)
Dropout	D 0.5
Flatten	
Dense	N dense1
Dropout	D 0.5
Dense	N dense2
Dropout	D 0.5
Dense	N 1

**Table 8:** Results for top five combinations of convolutional kernel size. The same values of convolutional kernel length were achieved with different sweep.

Length of Convolution Kernel			Classification Evaluation	
**First Layer**	**Second Layer**	**Third Layer**	**Accuracy (%)**	**Error (-)**
1	11	41	88.320	0.2930
1	11	31	88.278	0.2911
1	11	31	88.269	0.2911
1	21	61	88.240	0.2903
1	21	61	88.224	0.2903

**Table 9:** Results of top five combinations considering the number of neurons in dense layers and number of filters in convolutional layers.

No. Filters			No. Neurons		Classification Evaluation	
**First Layer**	**Second Layer**	**Third Layer**	**Fourth Layer**	**Fifth Layer**	**Accuracy (%)**	**Error (-)**
30	120	120	100	30	86.58	0.3349
120	500	120	30	100	86.51	0.3361
300	300	60	500	100	86.47	0.3330
120	120	120	100	100	86.45	0.3339
300	500	60	100	100	86.38	0.3386

**Table 10:** Results of training with different batch sizes.

Batch Size	Accuracy (%)	Error (-)
100	82.81	0.4183
250	82.58	0.4248
500	82.57	0.4252
1000	83.23	0.4004
2000	82.81	0.3973

**Table 11:** Testing the accuracy with different kernel size in the first layer of the proposed NN.

Kernel Size			Results	
**First Layer**	**Second Layer**	**Third Layer**	**ACC (%)**	**Error (-)**
1	15	45	88.11	0.2947
2	15	45	87.84	0.2988
3	15	45	87.76	0.2992

**Table 12:** Final structure of a convolutional neural network used for apnea and desaturation detection.

Layer Name	Parameter	No. of Weights	Data Size
2D CL	K (1 × 1) F 174	384	2 × 500 × 174
MaxPool	M (1,2)		2 × 250 × 174
Dropout	D 0.6		
2D CL	K (2 × 15) F 308	1,608,068	1 × 236 × 308
MaxPool	M (1,2)		1 × 118 × 308
Dropout	D 0.6		
2D CL	K (1 × 45) F 96	1,330,656	1 × 74 × 96
MaxPool	M (1,2)		1 × 37 × 96
Dropout	D 0.6		
Flatten			3552
Dense	N 148	525,844	148
Dropout	D 0.6		
Dense	N 86	12,814	86
Dropout	D 0.6		
Dense	N 1	87	1

**Table 13:** Results of classification by the proposed NN in the content of apnea segments under various thresholds.

Threshold (-)	Accuracy (%)	Sensitivity (%)	Specificity (%)
0.3	82.21	96.22	68.21
0.5	84.27	88.39	80.14
0.7	82.92	81.64	84.21
AUC (-)	0.9034		

**Table 14:** Results of classification by proposed NN in the content of desaturation segments under various thresholds.

Threshold (-)	Accuracy (%)	Sensitivity (%)	Specificity (%)
0.3	69.04	93.74	44.34
0.5	74.19	82.62	65.76
0.7	74.41	67.46	81.37
AUC (-)	0.8264		

**Table 15:** Results of classification by *k*-NN in content of apnea and desaturation segments under various *k*.

	Apnea	Desaturation
k **(-)**	**Accuracy (%)**	**Accuracy (%)**
5	83.06	55.77
20	84.22	57.74
50	84.03	59.11
100	83.71	59.93
200	82.81	60.78
500	80.96	61.61
1000	78.91	61.37

**Table 16:** Results of classification by *k*-NN in content of apnea (k=20) and desaturation (k=500) segments.

	Apnea	Desaturation
Accuracy (%)	83.39	64.33
Sensitivity (%)	88.31	54.05
Specificity (%)	78.47	74.61

## Sample Availability

GUI is available on github: https://github.com/mabartcz/ApnoeDetect, accessed on 16 November 2021. Complete codes of neural network design and its training and testing for raw data input are avaiable on github: https://github.com/mabartcz/ApnoeDetectRaw, accessed on 16 November 2021.

## Abbreviations

The following abbreviations are used in this manuscript:

AASM	American Academy of Sleep Medicine
AB	Adaptive boosting
AHI	Apnea hypopnea index
ANN	Artificial neural network
BA	Bootstrap aggregating
CNN	Convolutional neural network
DBN	Deep belief network
DFA	Detrended fluctuation analysis
DNN	Deep neural network
ECG	Electrocardiographic signals
EEG	Electroencephalographic signals
EMG	Electromyographic signals
EOG	Electrooculographic signals
*k*-NN	k-nearest neighbors
LSTM	Long short-term memory
NN	Neural network
OSAS	Obstructive sleep apnea syndrome
PCA	Principal component analysis
PO	Pulse oximetry
PSG	Polysomnography
RBM	Restricted Boltzmann machines
RF	Random forest
RNN	Recurrent neural network
RS	Respiratory signals
SAS	Sleep apnea syndrome
SLBP	Symmetrically weighted local binary patterns
SVM	Support vector machine
t-SNE	t-distributed stochastic neighbor embedding
